# Author Correction: Inhibitory neurotransmission drives endocannabinoid degradation to promote memory consolidation

**DOI:** 10.1038/s41467-021-21329-7

**Published:** 2021-02-08

**Authors:** Christophe J. Dubois, Jessica Fawcett-Patel, Paul A. Katzman, Siqiong June Liu

**Affiliations:** 1 Department of Cell Biology and Anatomy, LSU Health Sciences Center, New Orleans, LA 70112 USA; 2 Southeast Louisiana VA Healthcare System, New Orleans, LA 70119 USA; 3 Present Address: Univ. Bordeaux, CNRS, EPHE, INCIA, UMR 5287, 33000 Bordeaux, France

**Keywords:** Cellular neuroscience, Emotion, Consolidation, Synaptic plasticity

Correction to: *Nature Communications* 10.1038/s41467-020-20121-3, published online 17 December 2020.

The original version of this Article omitted the following from the Acknowledgements: Veterans Administration Grant BX003893.

This has now been corrected in both the PDF and HTML versions of the Article.

