# Supplementary material for: Inhibitory neurotransmission drives endocannabinoid degradation to promote memory consolidation
Source: Nat Commun. 2020 Dec 17;11:6407. doi: 10.1038/s41467-020-20121-3 (PMC7747732; doi:10.1038/s41467-020-20121-3)
Supplement: Supplementary file 1 — Supplementary Information [file 41467_2020_20121_MOESM1_ESM.pdf]

Supplementary Information for

Inhibitory neurotransmission drives endocannabinoid degradation to promote memory consolidation

Authors: Christophe J. Dubois, Jessica Fawcett-Patel, Paul A. Katzman and Siqiong June Liu

Affiliations: Department of Cell Biology and Anatomy, LSU Health Sciences Center, New Orleans, LA 70112 USA.

This PDF file includes:

Supplementary Figures 1 – 6
Supplementary Tables 1 – 4
Reagents and resource table

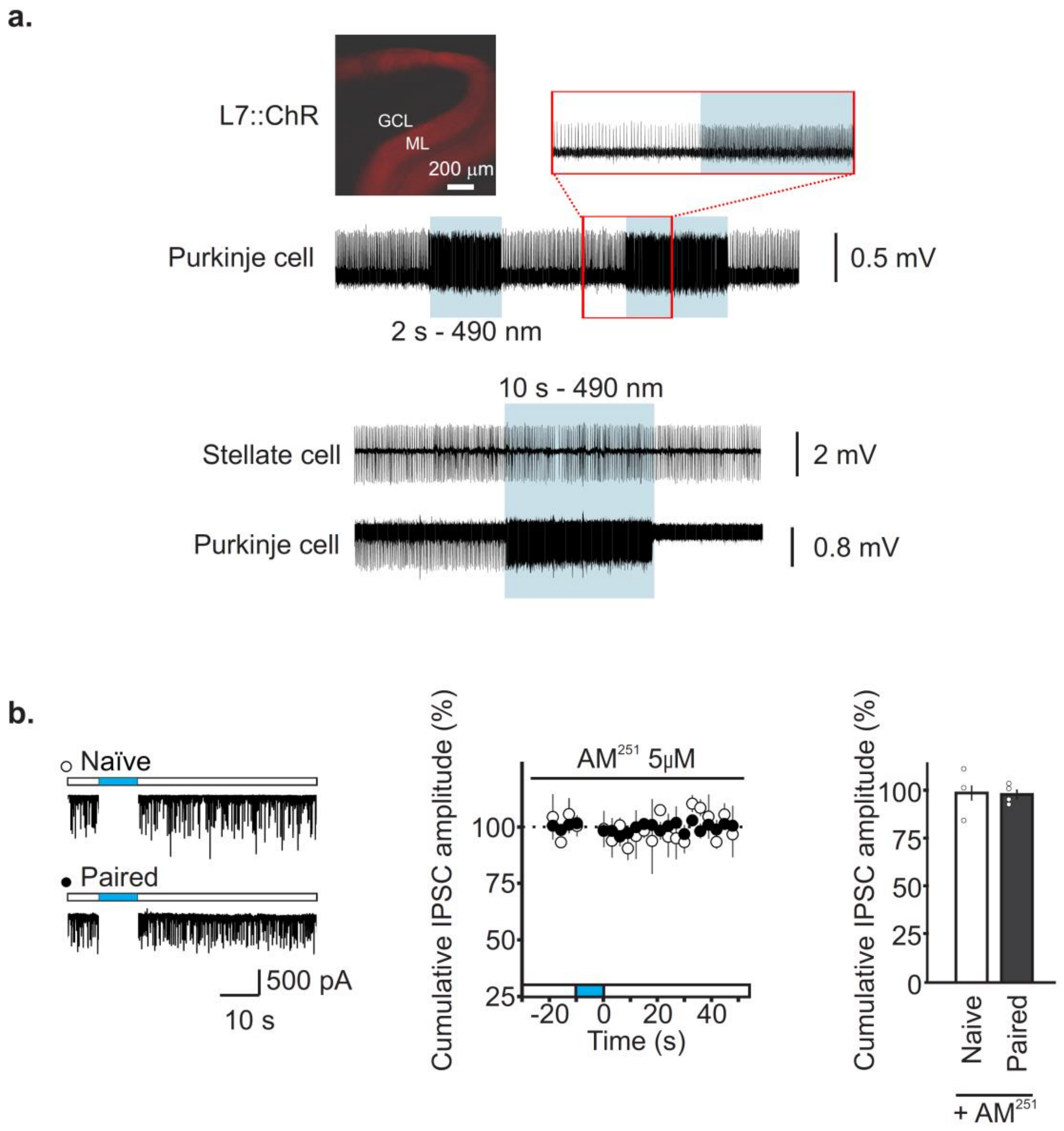

<u>Figure S1.</u> Related to figure 2. Optogenetic stimulation of Purkinje cells reduces spontaneous IPSCs in stellate cells via the activation of CB1Rs. (**a**) *Top left*, the expression of tomato-ChR in the cerebellar cortex of L7::ChR mice was restricted to Purkinje cells. Middle, action potential firing in a Purkinje cell was increased during photostimulation. Bottom, paired recording of action potentials in a stellate and a Purkinje cell from L7::ChR mouse. (**b**) Spontaneous IPSCs recorded in stellate cells from L7::ChR mouse. In the presence of the CB1R inverse agonist AM^{251} photostimulation of Purkinje cells for 10s failed to reduce sIPSCs. Examples (left), averaged time courses (center) and peak depression (right) of cumulative IPSC amplitude from SCs of naïve mice (n=3 cells, two-sided paired t-test, P>0.05) and conditioned mice (n=4 cells, two-sided paired t-test, P>0.05). Data are presented as mean values ± SEM. Statistical analysis and *P* values can be found in the statistical table (Supplementary Table 4) and original data in the Source Data file. The representative experiment in (**a**) has been independently replicated more than 3 times.

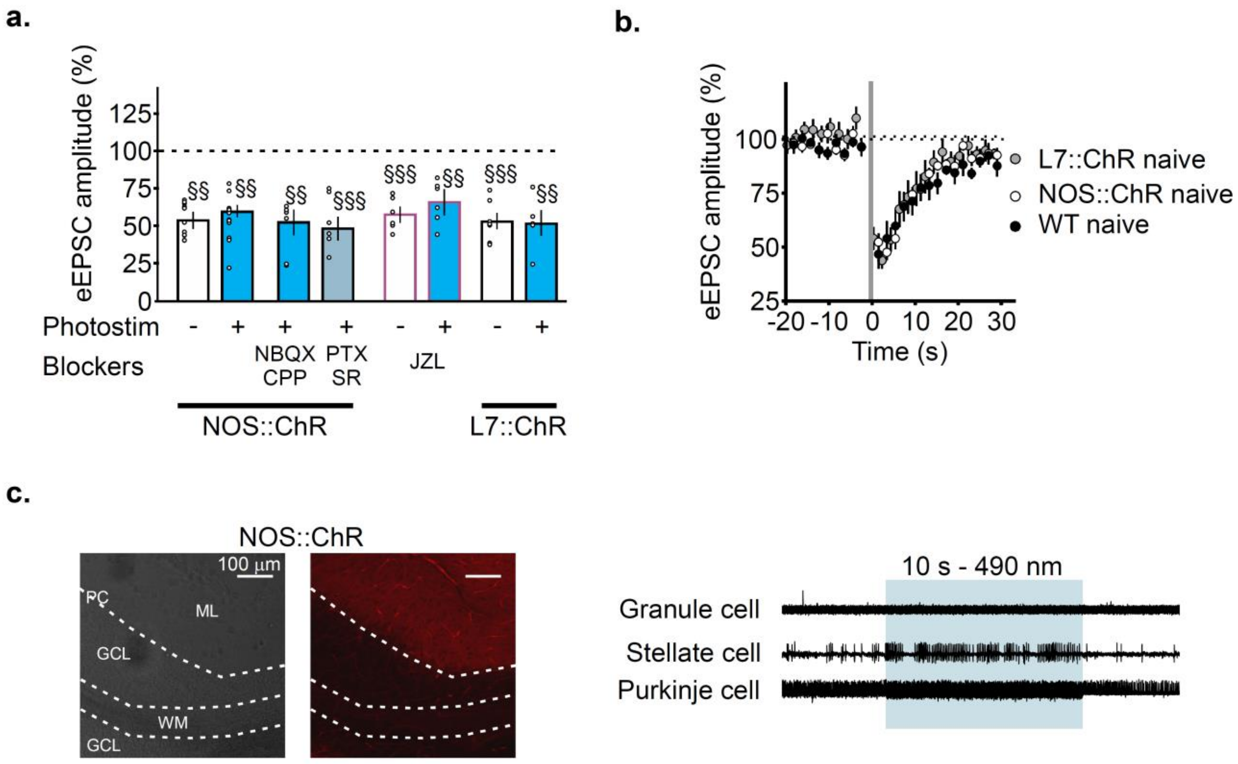

<u>Figure S2.</u> Related to figure 4. (**a**) Peak amplitude of DSE from experiments presented in Fig 4. (**b**) DSE in wild type naïve (n = 11 cells, black circles), non-stimulated NOS::ChR (n = 6 cells, open circles) and non-stimulated L7::ChR (n = 7 cells, grey circles) mice were not different. (**c**) Left, expression of tomato-ChR in the cerebellar cortex of NOS::ChR mice. Expression was restricted to GABAergic neurons. Right, typical extracellular recordings in granule, stellate and Purkinje cells showing an increased action potential firing in stellate and Purkinje cells, but not in granule cells, during photostimulation. Effect of each experimental condition on eEPSC amplitude after DSE in (**a**) was assessed with two-sided paired t-tests, §§, P<0.01, §§§, P<0.001. Data are presented as mean values ± SEM. Statistical analysis and *P* values can be found in the statistical table (Supplementary Table 4) and original data in the Source Data file. The representative experiment in (**c**) has been independently replicated more than 3 times.

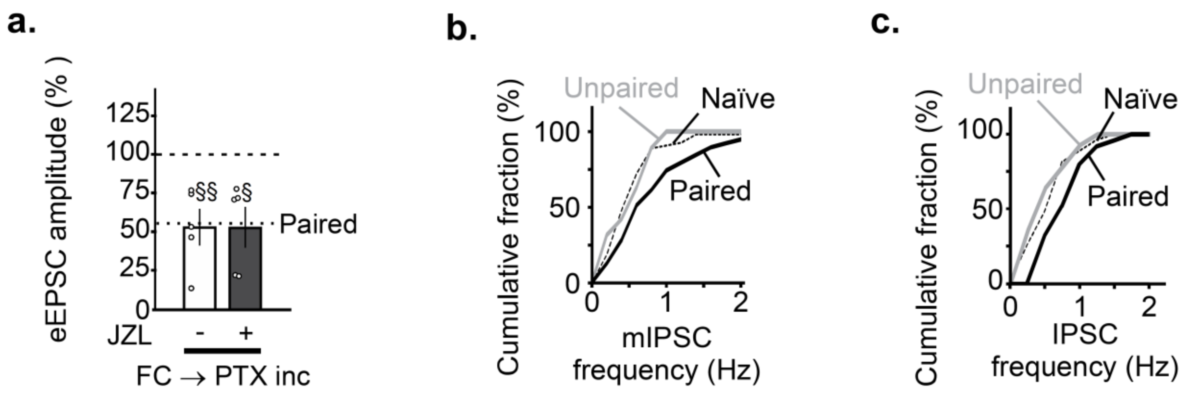

<u>Figure S3.</u> **Related to figure 5.** (**a**) Peak suppression of EPSCs from experiments presented in Fig 5. (**b**) mIPSCs were recorded in SCs. Cumulative fraction showing the distribution of mIPSC frequency (Naïve n=54 cells, UP n=19 cells, paired n=39 cells). (**c**) Cumulative fraction showing the distribution of spontaneous IPSC frequency (naïve n=27 cells, UP n=14 cells, paired n=25 cells). In (**a**) the effect of depolarization on eEPSC amplitude during DSE was assessed with two-sided paired t-tests §, *P*<0.05, §§, *P*<0.01. Data are presented as mean values ± SEM. Statistical analysis and *P* values can be found in the statistical table (Supplementary Table 4) and original data in the Source Data file.

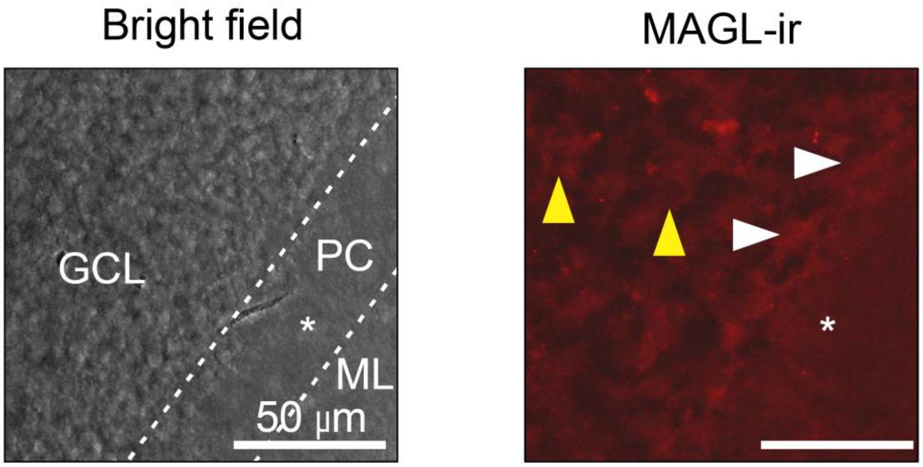

<u>Figure S4.</u> Related to figure 6. MAGL expression in the cerebellar cortex. Representative bright field and immunofluorescence for MAGL (MAGL-ir), independently replicated more than 3 times. MAGL-ir was found in the granule cell layer (GC) and molecular layers (ML), consistent with expression in granule cells (yellow arrows) and Bergman glial cells (white arrows) but not Purkinje cells (PC, *).

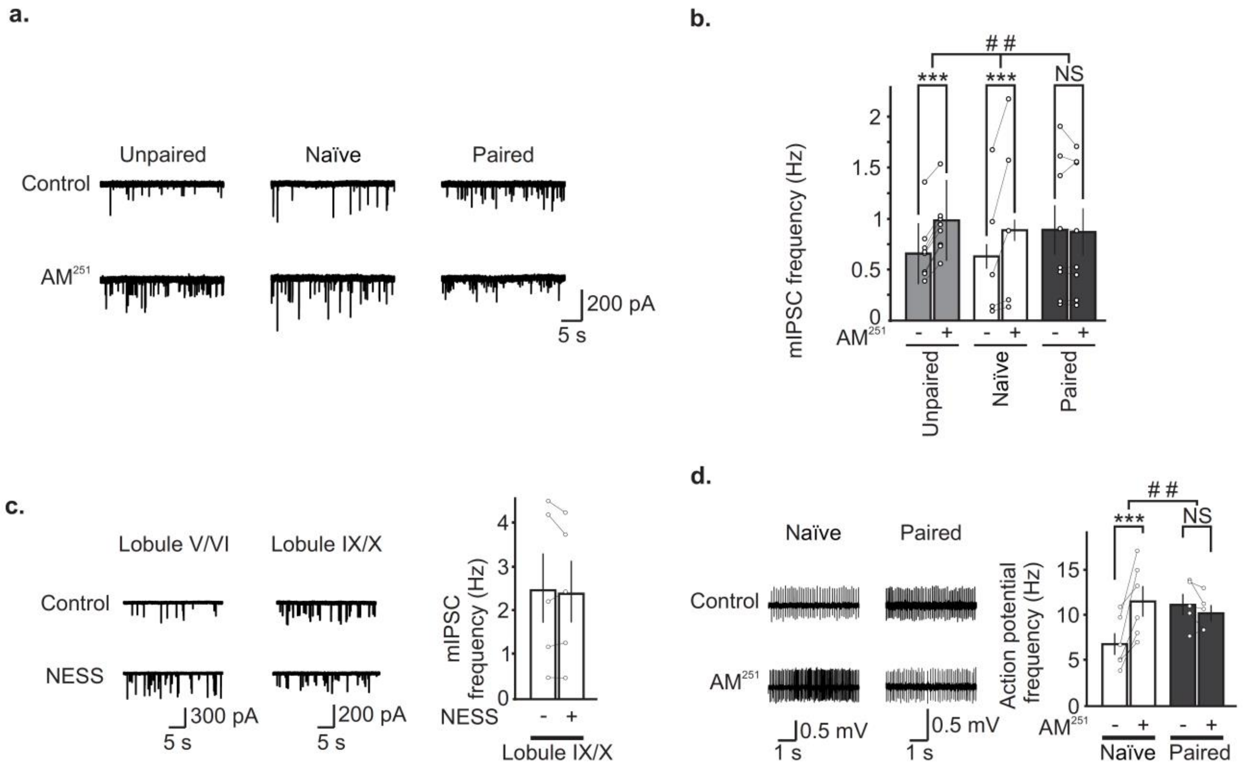

<u>Figure S5.</u> Related to figure 7. Fear conditioning abolishes the endogenous cannabinoid tone in cerebellar lobules V/VI. (**a**) Representative mIPSC recordings from stellate cells in lobules V/VI of naïve, unpaired control and conditioned mice before and during application of the CB1R inverse agonist AM^{251}. (**b**) Group data showing that AM^{251} increased mIPSC frequency in naïve (n=8 cells) and unpaired controls (n=5 cells), but not after fear conditioning (n=8 cells), indicating that tonic eCB signaling is present in controls but absent after fear conditioning. (**c**) Lack of tonic eCB signaling in lobules IX/X of naïve mice. Left and center, representative recordings from stellate cells in lobules V/VI (left) or lobules IX/X (center) of naïve mice before and during application of $NESS^{0327}$. Right, group data (n=5 cells). (**d**) The endogenous cannabinoid tone regulates action potential firing in stellate cells. Left, representative cell-attached recordings of action potential firing in stellate cells in the presence of inhibitory synaptic blockers with or without the CB1 inverse agonist AM^{251}. Right, Group data shows that AM^{251} increased action potential frequency in stellate cells from naïve (n=5 cells) but not conditioned mice (n=5 cells). Group comparisons in (**b**) and (**d**) were assessed by two-way ANOVAs with repeated measures (##, *P*=0.04 for mIPSC frequency and *P*=0.001 for action potential frequency) followed by Tukey post-hoc tests for drug effect within groups (***, *P*<0.001). In (**c**) the effect of NESS on mIPSC frequency was tested using a 2 sided paired t-test (*P*>0.05, see 7d). Data are presented as mean values ± SEM. Statistical analysis and *P* and *F* values can be found in the statistical table (Supplementary Table 4) and original data in the Source Data file.

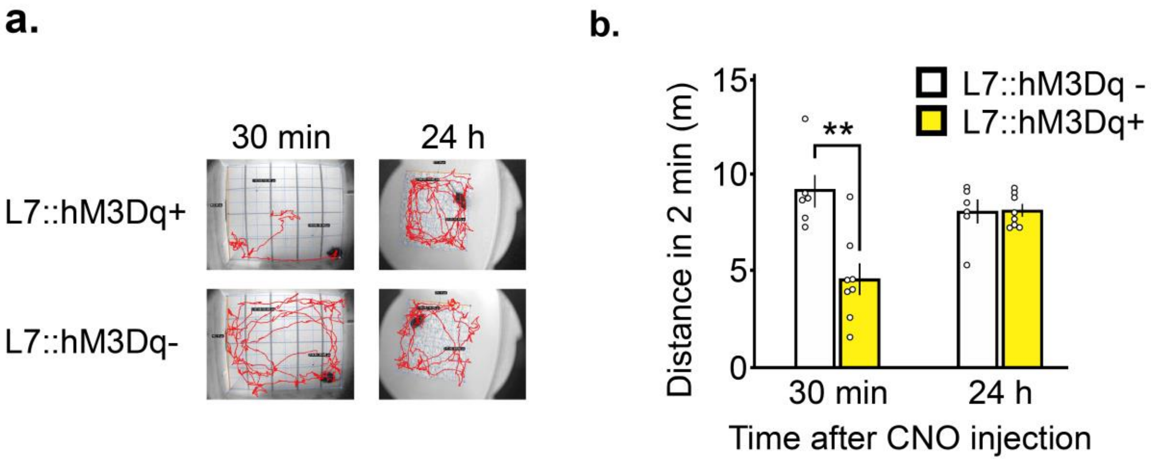

<u>Figure S6.</u> Related to figure 8. (**a**) Representative 2 min tracking of L7::hM3Dq+ (*top*) and L7::hM3Dq- (*bottom*) mice 30 min (*left*) and 24 hours (*right*) after CNO injection. (**b**) Group data of the average distance L7::hM3Dq+ (n=8 animals) and L7::hM3Dq- (n=6 animals) traveled during 2 min in a new environment, 30 min and 24 hours after CNO injection. The effect of CNO on the traveled distance was assessed with two-sided paired t-tests (**, P=0.002). Data are presented as mean values ± SEM. Statistical analysis and P values can be found in the statistical table (Supplementary Table 4) and original data in the Source Data file.

	L7::hM3Dq	L7::ChR	NOS::ChR
**Cerebellar cortex**	Purkinje cells	Purkinje cells	Purkinje cells<br>Molecular Layer interneurons<br>Blood vessels
**Whole brain**	Cerebellar Purkinje cells	NA	NA

<u>Table S1.</u> Summary of ChR-tomato or DREADD-mCitrine expression. For the L7::hM3Dq expression in the whole brain, both sagittal and coronal slices were examined. This table reflects a general observation and may not reflect scattered and rare ectopic expression as described by others [31].

Activation (%)	L7::hM3Dq	L7::ChR	NOS::ChR
**Purkinje cells**	100% (13/13)	100% (11/11)	100% (17/17)
**Stellate cells**	0% (0/4)	0% (0/13)<br>wc 0% (0/22)	77% (10/13)
**Basket cells**		0% (0/5)	
**Granule cells**			0% (0/2)

<u>Table S2.</u> Summary of the pharmacogenetic and optogenetic activation per cell type in the cerebellum. Rate of successful activation in extracellular recordings (or whole cell voltage clamp recordings, wc) either with CNO (DREADD) or blue light (ChR) (Number of cells activated over the number of cells recorded).

L7::hM3Dq genotype		-	+	-	+	-	+
**n**		**6**	**6**	**6**	**8**	**10**	**8**
**Drug injected**		**Saline**	**Saline**	**CNO**	**CNO**	**CNO + AM4113**	**CNO + AM4113**
**Fear conditioning**	**2 min habituation**	3 ± 3	11 ± 5	5 ± 1	5 ± 2	2 ± 2	2 ± 2
	**CS**	43 ± 8	40 ± 2	70 ± 4	64 ± 3	66 ± 4	60 ± 6
**Cued fear memory**	**2 min habituation**	7 ± 4	10 ± 5	4 ± 1	1 ± 0	10 ± 6	14 ± 6
	**CS**	60 ± 9	66 ± 11	58 ± 5	34 ± 4	67 ± 5	76 ± 2
**Contextual fear memory**	**2 min context**	N/A	N/A	34 ± 6	40 ± 8	45 ± 6	53 ± 4

<u>Table S3.</u> Summary of percentage freezing of L7::hM3Dq- and L7::hM3Dq+ mice during fear conditioning and memory retention testing.

Fig	Parameter analyzed	Conditions	n	Analysis	*F* value	*P* value
**1f**	τ of DSE	(N *vs* FC) x (ctrl *vs* JZL)		2 WAY ANOVA	*F* (1,30)= 4.936	**0.034**
		N *vs* FC without JZL	11 - 12	Tukey		**0.011**
		N *vs* FC with JZL	5 - 6	Tukey		0.423
		UP *vs* FC without JZL	6 - 12	Unpaired t-test		**<0.001**
**1g**	Amplitude eEPSC	UP (pre *vs* post depolarization)	6	Paired t-test		**<0.001**
		N (pre *vs* post depolarization)	11	Paired t-test		**<0.001**
		FC (pre *vs* post depolarization)	12	Paired t-test		**<0.001**
		N + JZL (pre *vs* post depolarization)	5	Paired t-test		**0.002**
		FC + JZL (pre *vs* post depolarization)	6	Paired t-test		**0.001**
		N + NESS (pre *vs* post depolarization)	5	Paired t-test		0.808
		FC + NESS (pre *vs* post depolarization)	5	Paired t-test		0.889
**2c**	τ of DSI	N *vs* FC	7 - 9	Unpaired t-test		**<0.001**
	Amplitude eIPSC	N (pre *vs* post depolarization)	7	Paired t-test		**<0.001**
		FC (pre *vs* post depolarization)	9	Paired t-test		**0.006**
		N *vs* FC (post depolarization)	7 - 9	Unpaired t-test		0.627
**2f**	τ of DSI	N *vs* FC	13 - 7	Unpaired t-test		**0.022**
	cumulative amplitude IPSC	N (pre *vs* post stim)	13	Paired t-test		**0.014**
		FC (pre *vs* post stim)	7	Paired t-test		**0.037**
		N *vs* FC (post stim)	13 - 7	Unpaired t-test		0.364
**3c**	mIPSC frequency	(N *vs* FC) x (pre *vs* WIN)		2 WAY RM ANOVA	*F* (1,12)= 8.143	**0.15**
		N (pre *vs* WIN)	7	Tukey		**0.028**
		FC (pre *vs* WIN)	7	Tukey		**<0.001**
	mIPSC amplitude	(N *vs* FC) x (pre *vs* WIN)		2 WAY RM ANOVA	*F* (1,12)= 1.206	0.292
		N (pre *vs* WIN)	7	Tukey		0.56
		FC (pre *vs* WIN)	7	Tukey		0.081
**3f**	current amplitude	(N *vs* FC) x (voltage steps)		2 WAY RM ANOVA	*F* (11,110)= 0.825	**0.615**
**4g**	τ of DSE	NOS::ChR (0x vs 8x Blue light) x (ctrl *vs* JZL)		2 WAY ANOVA	*F* (1,32)= 5.018	**0.032**
		NOS::ChR (0x vs 8x Blue light)	6 - 5	Unpaired t-test		**0.026**
		NOS::ChR (NBQX/CPP vs PTX/SR)	6 - 6	Unpaired t-test		**0.006**
		NOS::ChR (8x No drug vs PTX/SR)	5 - 6	Unpaired t-test		**0.007**
		NOS::ChR in JZL(0x vs 8x Blue light)	6 - 6	Unpaired t-test		0.875
		L7::ChR (0x vs 8x Blue light)	7 - 5	Unpaired t-test		0.611
		Indicative: NOS::ChR 8x Blue light<br>(No drug *vs* PTX/SR *vs* NBQX/CPP)		1 WAY ANOVA	*F* (2,16)= 13.252	**<0.001**
**5b**	$eIPSC_1$ amplitude	N *vs* FC	9 - 12	Unpaired t-test		**0.003**
		UP *vs* FC	9 - 12	Unpaired t-test		**0.006**
		N *vs* UP	9 - 9	Unpaired t-test		0.453
	PPR	N *vs* FC	9 - 12	Unpaired t-test		**0.002**
		UP *vs* FC	9 - 12	Unpaired t-test		**0.019**
		N *vs* UP	9 - 9	Unpaired t-test		0.154
**5d**	mIPSC frequency	N *vs* FC	54 – 39	Unpaired t-test		**0.006**
		UP *vs* FC	19 - 39	Unpaired t-test		**0.046**
		N *vs* UP	54 - 19	Unpaired t-test		0.695
	mIPSC Amplitude	N *vs* FC	54 – 39	Unpaired t-test		0.095
		UP *vs* FC	19 - 39	Unpaired t-test		0.586
		N *vs* UP	54 - 19	Unpaired t-test		0.11
**5f**	IPSC frequency	N *vs* FC	27 - 25	Unpaired t-test		**0.035**
		UP *vs* FC	14 - 25	Unpaired t-test		**0.019**
		N *vs* UP	27 - 14	Unpaired t-test		0.445
	IPSC Amplitude	N *vs* FC	27 - 25	Unpaired t-test		0.298
		UP *vs* FC	14 - 25	Unpaired t-test		0.514
		N *vs* UP	27 - 14	Unpaired t-test		0.794
**5i**	τ of DSE	(no PTX *vs* PTX) x (No JZL *vs* JZL)		2 WAY ANOVA	*F* (1,29)= 16.883	**<0.001**
		FC *vs* FC+PTX	12 - 5	Tukey		**<0.001**
		FC+PTX *vs* FC+PTX+JZL	5 - 5	Tukey		0.804
**6d**	MAGL-ir in the molecular layer	(UP vs FC) x (Lob V/VI vs Lob IX/X)		2 WAY RM ANOVA	*F* (1,39)= 7.79	**0.021**
		UP (Lob V/VI vs Lob IX/X)	10 - 10	Tukey		**0.008**
		FC (Lob V/VI vs Lob IX/X)	10 - 10	Tukey		0.625
		Lob V/VI (UP vs FC)	10 - 10	Tukey		**0.003**
		Lob IX/X (UP vs FC)	10 - 10	Tukey		0.117
	MAGL-ir in the granule cell layer	(UP vs FC) x (Lob V/VI vs Lob IX/X)		2 WAY RM ANOVA	*F* (1,39)= 0.407	0.551
		UP (Lob V/VI vs Lob IX/X)	10 - 10	Tukey		0.117
		FC (Lob V/VI vs Lob IX/X)	10 - 10	Tukey		0.469
		Lob V/VI (UP vs FC)	10 - 10	Tukey		0.64
		Lob IX/X (UP vs FC)	10 - 10	Tukey		0.867
**7a**	2-AG measurement	N vs UP vs FC		1 WAY ANOVA	*F* (2,13)= 8.574	**0.013**
		N vs FC	5 - 5	Tukey		**0.014**
		UP vs FC	4 - 5	Tukey		**0.045**
		N vs UP	5 - 4	Tukey		0.851
**7c**	mIPSC frequency	(N *vs* FC *vs* UP) x (pre *vs* NESS)		2 WAY RM ANOVA	*F* (2,39)= 4.144	**0.034**
		N (pre *vs* NESS)	9	Paired t-test		**<0.001**
		UP (pre *vs* NESS)	6	Paired t-test		**0.026**
		FC (pre *vs* NESS)	5	Paired t-test		0.869
	mIPSC amplitude (not shown)	(N *vs* FC *vs* UP) x (pre *vs* NESS)		2 WAY RM ANOVA	*F* (2,39)= 0.350	0.709
		N (pre *vs* NESS)	9	Paired t-test		0.178
		UP (pre *vs* NESS)	6	Paired t-test		0.784
		FC (pre *vs* NESS)	5	Paired t-test		0.878
**7d**	mIPSC frequency	N in lob IX/X (pre *vs* NESS)	5	Paired t-test		0.539
		N (pre *vs* AM^{251})	9	Paired t-test		**0.002**
		N (pre *vs* AM^{281})	6	Paired t-test		**0.044**
		UP (pre *vs* AM^{251})	5	Paired t-test		**0.048**
		FC (pre *vs* AM^{251})	8	Paired t-test		0.687
	mIPSC amplitude (not shown)	N in lob IX/X (pre *vs* NESS)	5	Paired t-test		0.073
		N (pre *vs* AM^{251})	9	Paired t-test		0.182
		N (pre *vs* AM^{281})	6	Paired t-test		0.307
		UP (pre *vs* AM^{251})	5	Paired t-test		0.343
		FC (pre *vs* AM^{251})	8	Paired t-test		0.11

Fig	Parameter analyzed	Conditions	n	Analysis	*F* value	*P* value
7f	mIPSC frequency	N (pre *vs* JZL$^{184/195}$)	7	Paired t-test		0.142
		FC (pre *vs* JZL$^{184/195}$)	9	Paired t-test		**0.008**
		FC (JZL$^{184/195}$ *vs* JZL$^{184/195}$+NESS^{0327})	5	Paired t-test		**0.039**
	mIPSC amplitude (not shown)	N (pre *vs* JZL$^{184/195}$)	7	Paired t-test		0.397
		FC (pre *vs* JZL$^{184/195}$)	9	Paired t-test		0.169
		FC (JZL$^{184/195}$ *vs* JZL$^{184/195}$+NESS^{0327})	5	Paired t-test		0.39
8c	IPSC frequency	(pre *vs* 30 min after CNO) x (No NESS vs NESS)		2 WAY RM ANOVA	*F* (1,17)= 10.005	**0.016**
		FC (pre *vs* 30 min after CNO)	5	Tukey		**0.002**
		FC in NESS (pre *vs* 30 min after CNO)	4	Tukey		0.873
	IPSC amplitude (not shown)	(pre *vs* 30 min after CNO) x (No NESS vs NESS)		2 WAY RM ANOVA	*F* (1,16)= 2.864	0.142
		FC (pre *vs* 30 min after CNO)	5	Tukey		0.486
		FC in NESS (pre *vs* 30 min after CNO)	4	Tukey		0.169
8e	Freezing	Saline injection L7::hM3Dq - vs + (cued)	6 - 6	Unpaired t-test		0.638
		L7::hM3Dq - (cued) Saline vs CNO	6 - 6	Unpaired t-test		0.477
		L7::hM3Dq + (cued) Saline vs CNO	7 - 6	Unpaired t-test		**0.024**
		CNO injection L7::hM3Dq - vs + (cued)	6 - 8	Unpaired t-test		**0.001**
		CNO injection L7::hM3Dq - vs + (contextual)	6 - 8	Unpaired t-test		0.583
9b	Freezing	L7::hM3Dq - vs + (cued)	12 - 9	Unpaired t-test		0.097
		L7::hM3Dq - vs + (contextual)	12 - 9	Unpaired t-test		0.25
		L7::hM3Dq + (cued) CNO vs CNO+ AM	8 - 9	Unpaired t-test		**<0.001**
10c	MAGL-ir in the molecular layer	CNO injection L7::hM3Dq - vs + (cued)	6 - 10	Unpaired t-test		**0.044**
10e	IPSC frequency	L7::hM3Dq - vs + (No drug)	5 - 5	Unpaired t-test		**0.044**
		L7::hM3Dq + (No drug vs NESS)	5 - 4	Unpaired t-test		**0.041**
S1b	cumulative amplitude IPSC	N (pre *vs* post stim) in AM^{251}	3	Paired t-test		0.765
		FC (pre *vs* post stim) in AM^{251}	4	Paired t-test		0.245
		N *vs* FC (post stim) in AM^{251}	3 - 4	Unpaired t-test		0.488
S2b	eEPSC Amplitude	NOS::ChR 0x BL (pre vs post depolarization)	6	Paired t-test		**0.004**
		NOS::ChR 8x BL (pre vs post depolarization)	5	Paired t-test		**0.006**
		NOS::ChR 8x BL NBQX/CPP (pre vs post depolarization)	6	Paired t-test		**0.006**
		NOS::ChR 8x BL in PTX/SR (pre vs post depolarization)	6	Paired t-test		**<0.001**
		L7::ChR 0x BL (pre vs post depolarization)	7	Paired t-test		**<0.001**
		L7::ChR 8x BL (pre vs post depolarization)	5	Paired t-test		**0.009**
S3a	eEPSC Amplitude	FC → PTX (pre *vs* post depolarization)	5	Paired t-test		**0.006**
		FC → PTX + JZL (pre vs post depolarization)	5	Paired t-test		**0.024**
S5b	mIPSC frequency	(N *vs* FC *vs* UP) x (pre *vs* AM^{251})		2 WAY RM ANOVA	*F* (2,43)= 7.346	**0.004**
		N (pre *vs* AM^{251})	9	Tukey		**<0.001**
		UP (pre *vs* AM^{251})	5	Tukey		**<0.001**
		FC (pre *vs* AM^{251})	8	Tukey		0.745
	mIPSC amplitude (not shown)	(N *vs* FC *vs* UP) x (pre *vs* AM^{251})		2 WAY RM ANOVA	*F* (2,43)= 0.268	0.768
		N (pre *vs* AM^{251})	9	Tukey		0.068
		UP (pre *vs* AM^{251})	5	Tukey		0.603
		FC (pre *vs* AM^{251})	8	Tukey		0.18
S5d	Action potential frequency	(pre *vs* AM^{251}) x (N *vs* FC)		2 WAY RM ANOVA	*F* (1,21)= 20.216	**0.001**
		N (pre *vs* AM^{251})	5	Tukey		**<0.001**
		FC (pre *vs* AM^{251})	5	Tukey		0.341
S6b	Distance	L7::hM3Dq - vs + (30 min)	6 - 8	Unpaired t-test		**0.002**
		L7::hM3Dq - vs + (24h)	6 - 8	Unpaired t-test		0.922

Table S4. Statistical table. Bold *P* values indicate a significant effect (P<0.05). N, Naive; UP, unpaired; FC, paired). RM ANOVA, repeated measures ANOVA.

REAGENT or RESOURCE	SOURCE	IDENTIFIER
Antibodies		
Goat polyclonal anti-MAGL	Everest Biotech	EB08850
Donkey anti-goat Dylight 549<br>Donkey anti-goat Cy3	Jackson<br>Immunoresearch	#705-505-147<br>#705-165-147
Chemicals, Peptides, and Recombinant Proteins		
**TTX** (Tetrodotoxin)	Ascent Scientific	Asc-055
**CPP** (3-((R)-2-Carboxypiperazin-4-yl)-propyl-1-phosphonic acid)	AbCam	Ab120159
**NBQX** (2,3-Dioxo-6-nitro-1,2,3,4-tetrahydrobenzo[f]quinoxaline-7-sulfonamide)	AbCam	Ab120046
**SR-95531** (6-Imino-3-(4-methoxyphenyl)-1(6H)-pyridazinebutanoic acid hydrobromide)	AbCam	Ab120042
**PTX** (Picrotoxin)	AbCam	Ab 120315
**AM**^{251} (N-(Piperidin-1-yl)-5-(4-iodophenyl)-1-(2,4-dichlorophenyl)-4-methyl-1H-pyrazole-3-carboxamide)	Cayman chemical company	#71670
**AM**^{281} (1-(2,4-Dichlorophenyl)-5-(4-iodophenyl)-4-methyl-N-4-morpholinyl-1H-pyrazole-3-carboxamide)	Tocris	#1115
**AM**^{4113} 5-(4-chlorophenyl)-1-(2,4-dichlorophenyl)-4-methyl-1H-pyrazole-3-carboxamide	Cayman chemical company	#20581
**NESS**^{0327} (8-chloro-1-(2,4-dichlorophenyl)-1,4,5,6-tetrahydro-N-1-piperidinyl-benzo[6,7]cyclohepta[1,2-c]pyrazole-3-carboxamide)	Cayman chemical company	#10004184
**WIN**$^{55,212-2}$ ((R)-(+)-[2,3-Dihydro-5-methyl-3-(4-morpholinylmethyl)pyrrolo[1,2,3-de]-1,4-benzoxazin-6-yl]-1-naphthalenylmethanone mesylate)	Ascent Scientific	Asc-085
**JZL**^{184} (4-[Bis(1,3-benzodioxol-5-yl)hydroxymethyl]-1-piperidinecarboxylic acid 4-nitrophenyl ester)	Enzo	BMLEI391-0010
**JZL**^{195} (4-[(3-Phenoxyphenyl)methyl]-1-piperazinecarboxylic acid 4-nitrophenyl ester)	AbCam	Ab144653
**CNO** (8-Chloro-11-(4-methyl-4-oxido-1-piperazinyl)-5H-dibenzo[b,e][1,4] diazepine)	RTI international	CAS#34233-69-7<br>NH# C-929
Experimental Models: Organisms/Strains		
C57Bl/6J WT	The Jackson lab	stock 000664
NOS::CRE (B6.129-Nos1<tm1(cre) Mgmj>/J)	The Jackson lab	Stock 017526
L7::CRE (B6.129-Tg(Pcp2-cre)2Mpin/J)	The Jackson lab	stock 004146
floxed ChR (B6.Cg-Gt(ROSA)26Sortm27.1(CAG-COP4*H134R/tdTomato) Hze/J)	The Jackson lab	stock 012567
floxed Gq-DREADD (Gt(ROSA)26Sortm2 (CAG-CHRM3*,-mCitrine)Ute/J)	The Jackson lab	stock 026220
Software and Algorithms		
Frame by frame analysis software	Liu et al., 2010	

Resource table
